# Biomass allometric equation and expansion factor for a mountain moist evergreen forest in Mozambique

**DOI:** 10.1186/s13021-018-0111-7

**Published:** 2018-11-26

**Authors:** Sá Nogueira Lisboa, Benard Soares Guedes, Natasha Ribeiro, Almeida Sitoe

**Affiliations:** Department of Forestry, Faculty of Agronomy and Forestry Engineering, Eduardo Mondlane University, Main Campus, Building # 1, P.O.Box 257, Maputo, Mozambique

**Keywords:** Above-ground tree biomass, Carbon stock, Pan-tropical equation, Biomass expansion factor

## Abstract

**Background:**

Worldwide, forests are an important carbon sink and thus are key to mitigate the effects of climate change. Mountain moist evergreen forests in Mozambique are threatened by agricultural expansion, uncontrolled logging, and firewood collection, thus compromising their role in carbon sequestration. There is lack of local tools for above-ground biomass (AGB) estimation of mountain moist evergreen forest, hence carbon emissions from deforestation and forest degradation are not adequately known. This study aimed to develop biomass allometric equations (BAE) and biomass expansion factor (BEF) for the estimation of total above-ground carbon stock in mountain moist evergreen forest.

**Methods:**

The destructive method was used, whereby 39 trees were felled and measured for diameter at breast height (*DBH*), total height and the commercial height. We determined the wood basic density, the total dry weight and merchantable timber volume by Smalian’s formula. Six biomass allometric models were fitted using non-linear least square regression. The BEF was determined based on the relationship between bole stem dry weight and total dry weight of the tree. To estimate the mean AGB of the forest, a forest inventory was conducted using 27 temporary square plots. The applicability of Marzoli’s volume equation was compared with Smalian’s volume equation in order to check whether Marzoli’s volume from national forest inventory can be used to predict AGB using BEF.

**Results:**

The best model was the power model with only *DBH* as predictor variable, which provided an estimated mean AGB of 291 ± 141 Mg ha^−1^ (mean ± 95% confidence level). The mean wood basic density of sampled trees was 0.715 ± 0.182 g cm^−3^. The average BEF was of 2.05 ± 0.15 and the estimated mean AGB of 387 ± 126 Mg ha^−1^. The BAE from miombo woodland within the vicinity of the study area underestimates the AGB for all sampled trees. Chave et al.’s pantropical equation of moist forest did not fit to the Moribane Forest Reserve, while Brown’s equation of moist forest had a good fit to the Moribane Forest Reserve, having generated 1.2% of bias, very close to that generated by the selected model of this study. BEF showed to be reliable when combined with stand mean volume from Marzoli’s National Forestry Inventory equation.

**Conclusion:**

The BAE and the BEF function developed in this study can be used to estimate the AGB of the mountain moist evergreen forests at Moribane Forest Reserve in Mozambique. However, the use of the biomass allometric model should be preferable when *DBH* information is available.

## Introduction

Forests generally, and moist tropical forests specifically, have huge amounts of carbon in their biomass [1]. This means that tropical forest vegetation, which accounts for about 50% of the world’s forest, store not less than 46% of the world’s living terrestrial carbon pool, and the tropical soils store about 11.55% of the world’s soil carbon pool [2, 3]. Mountain moist evergreen forests cover a small part (less than 3%) of the total forest area in Mozambique (400,680 km^2^) [4], and typical examples can be found in the Chimanimani outskirts, where Moribane Forest Reserve (MFR) is located [4, 5]. In fact, the MFR is among the largest moist evergreen forests in Mozambique [4]. The flora of these forests is still poorly known, but includes some of the locally threatened or endemic plant species [6].

In spite of still containing high species richness and diversity of plants and animals [6, 7], the MFR is at risk of deforestation and forest degradation (D&FD) [8]. Therefore, disturbance and land use can thus have large impacts on carbon emission into the atmosphere [1]. A case study carried in the Manica province, which included the MFR area, has observed annual biomass and carbon losses of 3.1% (2007–2010), attributable equally to D&FD [8]. The D&FD has resulted in negative impacts on biodiversity conservation and climate change [6, 9].

Solutions to reverse or slow down D&FD in MFR include improving forest carbon storage, protecting biodiversity, and sustaining livelihoods of forest-dependent people. This includes the implementation of emerging carbon credit market mechanisms such as Reducing Emissions from Deforestation and Forest Degradation (REDD+) [10]. Mountain moist evergreen forests have a great potential for conservation within the REDD+ context because they store large amounts of carbon, they have high biodiversity level and socio-ecological value [7, 8].

The Paris Agreement encourages developing countries to contribute to climate change mitigation by reducing emissions from deforestation, forest degradation, conserving carbon stocks, managing forest sustainably and enhancing forest carbon stocks [11]. With the REDD+ centered on results-based mechanism, where carbon is the most important result indicator, the need to establish appropriate allometric models and biomass expansion factor (BEF) has grown. Although information on biomass allometric equations (BAE) has been developed for African forests, little has been done for moist evergreen forests, particularly those in Mozambique [12].

So, for countries that need to implement the REDD+ mechanism, it is important to develop biomass local models and parameters per forests types to estimate accurately the greenhouse gas emissions from D&FD [13]. Appropriate BAE and BEF and reliable forest inventory data on biomass is essential to accurately quantify, monitor and report the impacts or benefits of REDD+ activities on climate change mitigation [13, 14]. BEF has been particularly useful because need aggregated data (mostly mean stand volume) from forest inventories in order to estimate average above-ground tree biomass. Therefore, it becomes very useful when stand volume of forest inventory is available but not only the individual trees as required by allometric model [14].

However, BAE and BEF functions previously developed in Mozambique were developed for forest types other than moist evergreen forest, e.g. lowland miombo woodland [15–17], mangrove forests [18] and mecrusse woodlands [19–21]. Moreover, the degree of reliability of the existing general allometric models and BEF functions and those suggested for moist in tropical zones [2, 22–24] must be checked if applied in a site different than that where they were originally developed [25].

In this study, we develop a pioneer BAE and a BEF for estimating total (stem, branches and foliage) above-ground tree biomass (AGB) of mountain moist evergreen forest in MFR, in central Mozambique. BEF function is intended to estimate total AGB using the wood volume and wood basic density [14, 22], which are provided by the national forest inventory [4].

## Materials and methods

### Study area

**Fig. 1:**
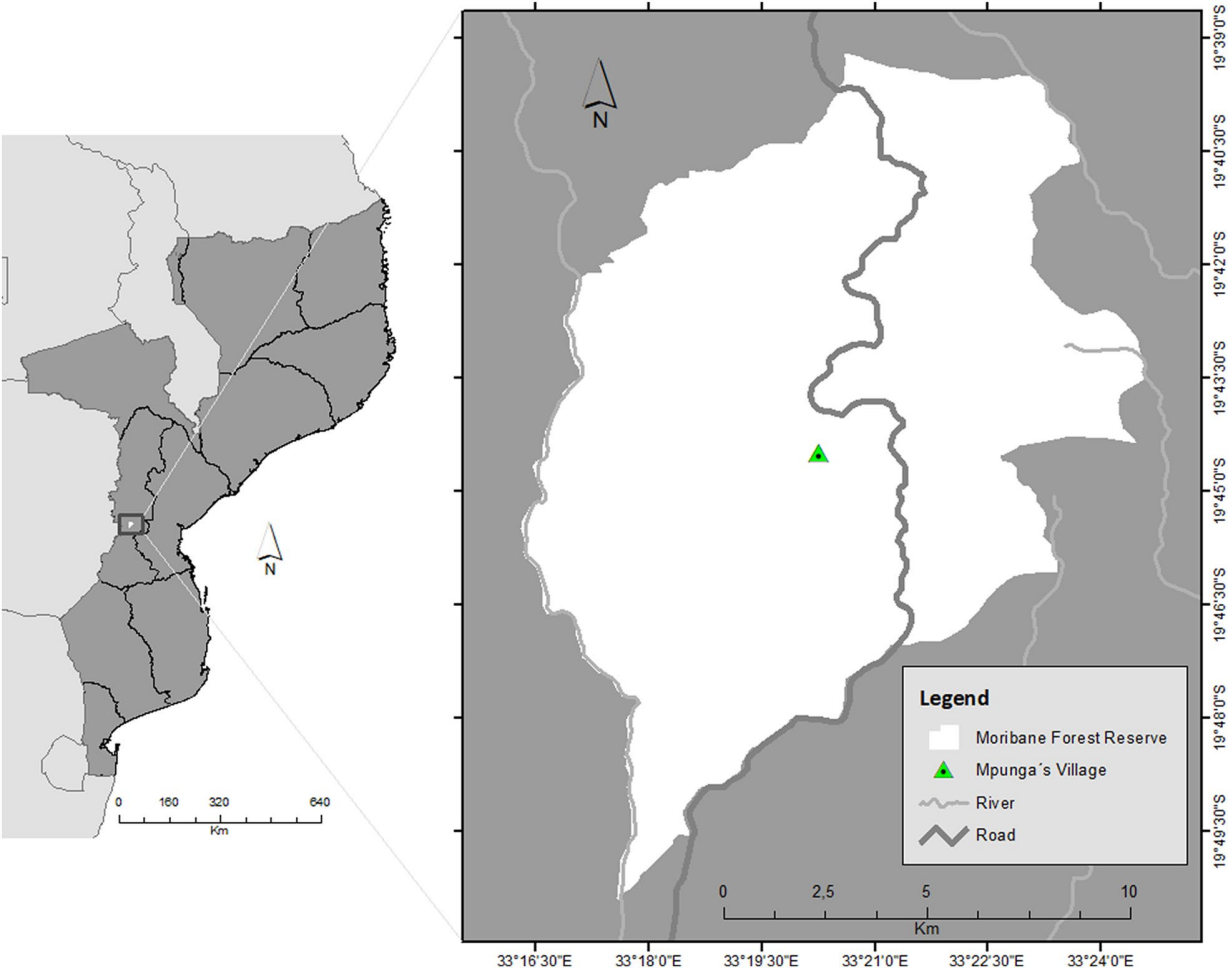
Geographical location of the MFR in the central province of Manica, Mozambique. The green dot (right) indicates the location of the main camping site of the reserve

This study was carried out at Moribane Forest Reserve (MFR), located in the district of Sussundenga in central Mozambique (S 19° 45′, 33° 22′ E) (Fig. 1). The MFR has a total area of about 53 km^2^ and was proclaimed as a conservation area in 1957 [26]. Since 2000, the MFR is part of the Chimanimani Transfrontier Conservation Area, which involves Mozambique and Zimbabwe. Extensive forest perturbance was caused in some parts of the forest by a devastating fire which occurred in 1992, subsequent to a very severe drought [6]. Sussundenga district had a population of 168 thousand in 2017 [27]. Artisanal mining in the highlands, deforestation for slash-and-burn agriculture, illegal hunting and logging are the main threats of biodiversity loss in MFR [28]. Ryan et al. [8] stated that within the MFR, biomass was lost at a rate of 2.8 ± 1.9% per year, with stocks changing from 19.4 ± 0.9 TgC in 2007 to 17.6 ± 0.9 TgC in 2010. Small-scale agriculture was the direct cause of 46 ± 17% of the total biomass loss, followed in magnitude by construction and miscellaneous activities (24 ± 11%), charcoal production (18 ± 9%), logging (9 ± 5%) and commercial agriculture (3 ± 2%) [8].

The moist evergreen forest is the main forest type of MFR, which is dominated by evergreen tree species and deciduous tree species, such as *Newtonia buchananii* (Baker) Gilbert & Boutique (Leguminosae), *Pteleopsis myrtifolia* (M. A. Lawson) Engl. & Diels (Combretaceae), *Millettia stuhlmannii* Taub. (Leguminosae), *Albizia gummifera* (J. F. Gmel.) C. A. Sm. (Leguminosae), *Khaya anthotheca* (Welw.) C. DC., among others [4, 6, 26, 29, 30]. The climate is classified as tropical, modified by altitude, according to Köppen climate classification [31]. The altitude range is 400–550 m a.s.l., the mean annual temperature range is 17–24 °C [30]. The mean annual rainfall range is 1200–1400 mm [29], and the soils range from sandy loam to sandy clay [32].

### Forest inventory and assessment of total above-ground tree biomass

A conventional forest inventory was carried on 27 square non-permanent inventory plots, 50 × 50 m (0.25 ha). At each sampling plot, diameter at breast height (*DBH* at 1.3 m from the ground), commercial tree height (HC), and total tree height (*TH*) were measured on all trees and shrubs with DBH ≥ 5 cm. All trees were identified at species level in the field by a skilled botanist. Total (stem, branches and foliage) AGB was estimated using the destructive method. Field sampling resulted in a total of 39 trees (Table 1), which were used to construct the BAE for the MFR. The trees for destructive sampling were selected randomly in the landscape considering the need to represent size classes of DBH from 5 to 60 cm. However, the national legislation does not allow any logging activities inside conservation areas in Mozambique. With this limitation, the sample trees were cut in the buffer zone of MFR, where human disturbance is high. Hence, some trees sized between 40 and 50 cm were not sampled.

**Table 1:** Descriptive statistics of the trees sampled used to construct the BAE and to determine the biomass expansion factor at the MFR in Mozambique

Variables and parameters	Description		
	Mean	SE	Range
DBH (cm)	21.46	12.84	5.50–57.00
Total tree height (m)	12.26	4.43	4.72–23.80
Commercial tree height (m)	5.14	2.77	1.40–10.60
Smalian’s stem volume (m^3^)	0.27	0.36	0.0072–1.58
Wood basic density (g cm^−3^)	0.71	0.16	0.46–0.91
Total dry weight (kg tree^−1^)	523.12	843.42	3.96–3539.06

*SE* is standard error of the mean

The DBH of the selected trees was measured using a caliper, whereas the commercial and total tree height/length were measured using a conventional measuring tape after the tree has been fallen. Each sampled tree was divided into three components (i) bole stem as the merchantable section, (ii) branches with minimum diameter of 3 cm, and (iii) twigs and leaves. The total fresh weight of each component was determined immediately in the field, using a mechanical weighing scale (max. 200 kg, precision 100 g). For each component, sub-samples weighing 200–3000 g were taken and their fresh weight determined in the field using a digital weighing scale (max. 3 kg, precision 0.5 g).

In the field, the stem of each sampled tree was measured according to the Smalian’s method for accurate stem volume estimation [33]. The stem of each individual tree was subdivided into billets, and the top end and the lower end of each section was measured for diameter using a caliper. The bole was divided in the following sections: 0.1, 0.4, 0.7, 1, 1.3 and 2 m length and then followed by intervals of 1 m until reaching the starting point of canopy of tree. Table 1 shows the descriptive statistics of the sampled trees.

### Laboratory measurements

The dry weight of all sub-samples (belonging to bole stem, branches and foliage) was determined in the laboratory after drying at 75 °C in an oven to constant weight. The dry weight of each sub-sample was recorded in the laboratory using a digital weighing scale (max. 3 kg, precision 0.5 g). Dry to fresh weight ratio of the sub-samples of each tree component was used to determine the dry weight of each tree component. The dry weight of tree components was added together to give the tree total above-ground dry weight (bole stem, branches, and foliage).

After determining the dry weight of each sub-sample of the bole stems, the subsamples were subjected to an additional treatment, in order to determine wood basic density (*WBD*). The determination of *WBD* was adapted from the procedure described in Malimbwi et al. [34] and Munishi and Shear [35]. From each stem sub-sample, four square specimens of 3 × 3 cm (with the height varying from 3 to 4 cm depending on the thickness of the stem disk sample) were extracted. Each specimen was submerged in water for a week in order to attain its fresh volume. Each specimen was then submerged in a graduated 1-l container with the precision of 0.1 cm^3^ for the estimation of the subsample volume. The *WBD* of each specimen was obtained by dividing its dry mass (in grams) by its corresponding wet volume (in cubic centimeters) [22, 36]. Finally, the *WBD* of the stem (g cm^−3^), which is shown in Table 1, was calculated averaging the sum of the ratio dry weight (sdw_*i*_; g) and the volume (sv_*i*_; cm^−3^) of each of the four specimens was extracted per sub-sample, using Eq. 1.

1 $$ WBD = \frac{1}{4}\mathop \sum \limits_{4}^{i = 1} \frac{{sdw_{i} }}{{sv_{i} }} $$

### Data analysis

#### Construction of biomass allometric equation and height–diameter regression models

Six alternative non-linear regression functions were selected as candidate BAE and tested in this study (Table 2). The adequacy of *DBH* alone (model 4), *DBH* in combination with total height (*TH*) (model 1 and 6), *DBH* in combination with WBD (model 3) and the combination between *DBH*, *TH* and *WBD* (model 2 and 5) as predicting variables of total dry weight (*tDW*) of standing forest (live trees) were selected as candidate BAE and tested in this study. We also tested the relationship between tree *TH* and *DBH*. The diameter–height models were selected from Ngomanda et al. [12] (model 7 is a power function and model 8 is Mitscherlisch function) and Mugasha et al. [37] (model 9 and 10), and were fitted using non-linear regression.

**Table 2:** Alternative models tested for predicting total above-ground tree biomass (model 1–6) and for predicting total height (model 7–10) in mountain moist evergreen forest of Moribane forest reserve in Mozambique

Model	Expression	Source
*Biomass allometric models*		
1	$tDW = b_{0} \times DBH^{{b_{1} }} \times TH^{{b_{2} }}$	Magalhães and Seifert [20]
2	$tWD = b_{0} \times DBH^{{b_{1} }} \times TH^{{b_{2} }} \times WD^{{b_{3} }}$	Ngomanda et al. [12], Chave et al. [42], Mugasha et al. [37]
3	$tDW = b_{0} \times DBH^{{b_{1} }} \times WD^{{b_{2} }}$	Mate et al. [15]
4	$tDW = b_{0} \times \left( {DBH} \right)^{b1}$	Guedes et al. [17]
5	$tDW = b_{0} \times (DBH^{2} \times TH \times WD)^{{b_{2} }}$	Ngomanda et al. [12], Chave et al. [42], Mugasha et al. [37]
6	$tDW = b_{0} \times \left( {DBH^{2} \times TH^{b1} } \right)$	
*Diameter–height models*		
7	$TH = 1.3 + b_{0} \times \left[ {{ \exp }\left( { - b_{1} \times \exp \left( { - b_{2} \times DBH} \right)} \right)} \right]$	Mugasha et al. [37]
8	$TH = b_{0} - b_{1} \times exp\left( { - b_{2} \times DBH} \right)$	Ngomanda et al. [12]
9	$TH = 1.3 + \left[ {DBH^{2} /b_{0} + b_{1} \times DBH + b_{2} \times DBH^{2} } \right]$	Mugasha et al. [37]
10	$TH = b_{0} \times DBH^{{b_{1} }}$	Ngomanda et al. [12]

*tDW* is total (stem, branches and foliage) dry weight of individual tree (kg tree^−1^)

*DBH* is diameter at breast height (cm)

*TH* is total height (m)

*WBD* is wood basic density (g cm^−3^)

*b*_0_, *b*_1_, *b*_2_ and *b*_3_ are the regression parameters

#### Model selection and evaluation criteria

The BAE and the height–diameter relationship model which showed the lowest value of residual standard error (RSE) and Akaike’s information criterion (AIC, Eq. 2) was chosen [18, 37, 38, 44]. Both BAE and height–diameter models were develop in R software, version 3.3.2 [40], using the non-linear least square regression approach in the ‘nlstools’ package [39]. For further analysis, we computed two other parameters, as suggested by Kachamba et al. [44], i.e. mean prediction error (MPE, Eq. 3), relative mean prediction error (RMPE, Eq. 4) for all alternative models tested in this study.

2 $$ AIC = n\text{Log}\left( {\mathop \sum \limits_{i = 1}^{n} \frac{{\left( {tDW_{est,i} - tDW_{obs,i} } \right)^{2} }}{n}} \right) + 2p $$

3 $$ MPE = \frac{{\left( {tDW_{est,i} - tDW_{obs,i} } \right)}}{n} $$

4 $$ RMPE = \mathop \sum \limits_{i = 1}^{n} \frac{MPE}{{\bar{Y}}} \times 100 $$

where AIC (unite less) is Akaike’s information criterion, MPE is mean prediction error (kg tree^−1^), RMPE is relative mean prediction error (%), *tDW*_est,*i*_ and *tDW*_*obs*,*i*_ are estimated and observed total dry weight of individually weighed tree *i*, respectively, $\overline{Y}$ is average of observed total dry weight (kg tree^−1^), *n* is total number of sampled trees, and *p* is number of parameters in the tested model. All model goodness-of-fit testing were performed in R software, version 3.5.1 [40].

#### Comparison with existing regression models

Finally, we compared the performance of prediction of our best BAE developed in this study with the BAE shown in Table 3, which were selected from the literature. We tested three moist forest models of Pan-tropical [22, 41, 42], one rainforest model from Tanzania [43], three lowland miombo woodland, of which one from Tanzania [37] and another model from Mozambique [17]. The comparison was made based on MPE and RMPE [17, 44]. The best model was considered to be the one that yielded the lowest MPE and RMPE value close to our selected biomass model.

**Table 3:** Models with diameter at breast height (*DBH*), total height (*TH*) and wood basic density (*WBD*) as independent variables selected from the literature and used to compare with the predictive accuracy of the BAE developed in this study

ID no.	Biomass allometric equation	Source	*DBH* range (cm)	Sampled trees	Forest type	Country
1	*tDW* = exp(− 2.134 + 2.530 × ln(DBH))	Brown [22]	5–148	170	Moist forest	Pan-tropical
2	*tDW* = exp(− 2.289 + 2.649 × ln (*DBH*)) − 0.021 × ln(*DBH*^2^))^*^	Pearson et al. [41]	5–148	170	Moist forest	Pan-tropical
3	*tDW *= 0.1754 × *DBH*^*2*.3238^	Guedes et al. [17]	5–53	155	Miombo woodland	Mozambique
4	*tDW* = 0.1014 * (WBD * *DBH*^2 ^* *TH*)^0.9510^	Mugasha et al. [37]	5–100	60	Miombo woodland	Tanzania
5	*tDW* = 0.0673 * (WBD * *DBH*^2 ^* *TH*)^0.976^	Chave et al. [42]	NA	4004	Moist forest	Pan-tropical
6	*tDW *= 0.1027 * *DBH*^*2*.4798^	Masota et al. [43]	5–100	60	Rainforest	Tanzania

*NA* not available

#### Biomass expansion factor

The BEF was calculated as the average ratio between total dry weight and total stem weight of all harvested trees using Eq. 5 [2, 14, 22, 23].

5 $$ BEF = \frac{1}{n} \times \mathop \sum \limits_{i = 1}^{n} \frac{{tDW_{i} }}{{tSW_{i} }} $$

where *BEF* (unit less) is biomass expansion factor, *tDW,*_*i*_ (kg tree^−1^) is total (bole stem, branches and foliage) dry weight of each individually sampled tree, *tSW,*_*i*_ (kg tree^−1^) is total dry weight of the bole stem alone and of each individually sampled tree, and *n* is the total number of sampled trees.

#### Stem volume

The stem volume was calculated by using two procedures: (i) the destructive method, i.e. the volume calculated per section using the Smalian’s formula (Eq. 6) as used by Henry et al. [45] in a similar study in a moist evergreen forest in Ghana; and (ii) considering the general factor form (Eq. 7), as suggested by Marzoli [4], which is the conventional procedure used to calculate stem volume (merchantable volume) in national forest inventories of native forests in Mozambique. We seek to understand the implication of using the national volume equation from Marzoli [4] against that generated in this study by the sectional method. The Marzoli [4]’s equation was used to predict mean volume per hectare from forest inventory data which was then used for prediction of AGB using BEF.

6 $$ V_{c} = \mathop \sum \limits_{i = 1}^{n} \left( {\frac{{D_{u}^{2} + D_{b}^{2} }}{8} \times L_{i} } \right) $$

7 $$ V_{c} = \frac{{\pi \times DBH^{2} }}{4} \times h_{c} \times f_{c} $$

where *V*_*c*_ (m^3^) is stem volume (otherwise merchantable volume), *DBH* (cm) is the diameter at breast height of all sampled trees, *D*_*b*_ (cm) is diameter of the lower cross-section, *D*_*u*_ (cm) is that of the upper cross-section; *L* (m) is length of stem*; h*_*c*_ (m) is tree commercial height, and *f*_*c*_ (0.8) is form factor for merchantable stem volume.

Since the equation suggested by Marzoli [4] takes a constant form factor (*f*_*c*_) for commercial height of trees, regardless of forest type and tree species differences, in this study we compared the stem volume calculated from the two procedures, under the specific conditions of the moist forest of MFR and using the Wilcoxon’s test (α = 0.05). By using Marzoli [4]’s equation, we aimed at evaluating the potential to use the BEF combined with the volume tables of the national forest inventory for quick estimates of AGB.

#### Estimations of total above-ground tree biomass

**Fig. 2:**
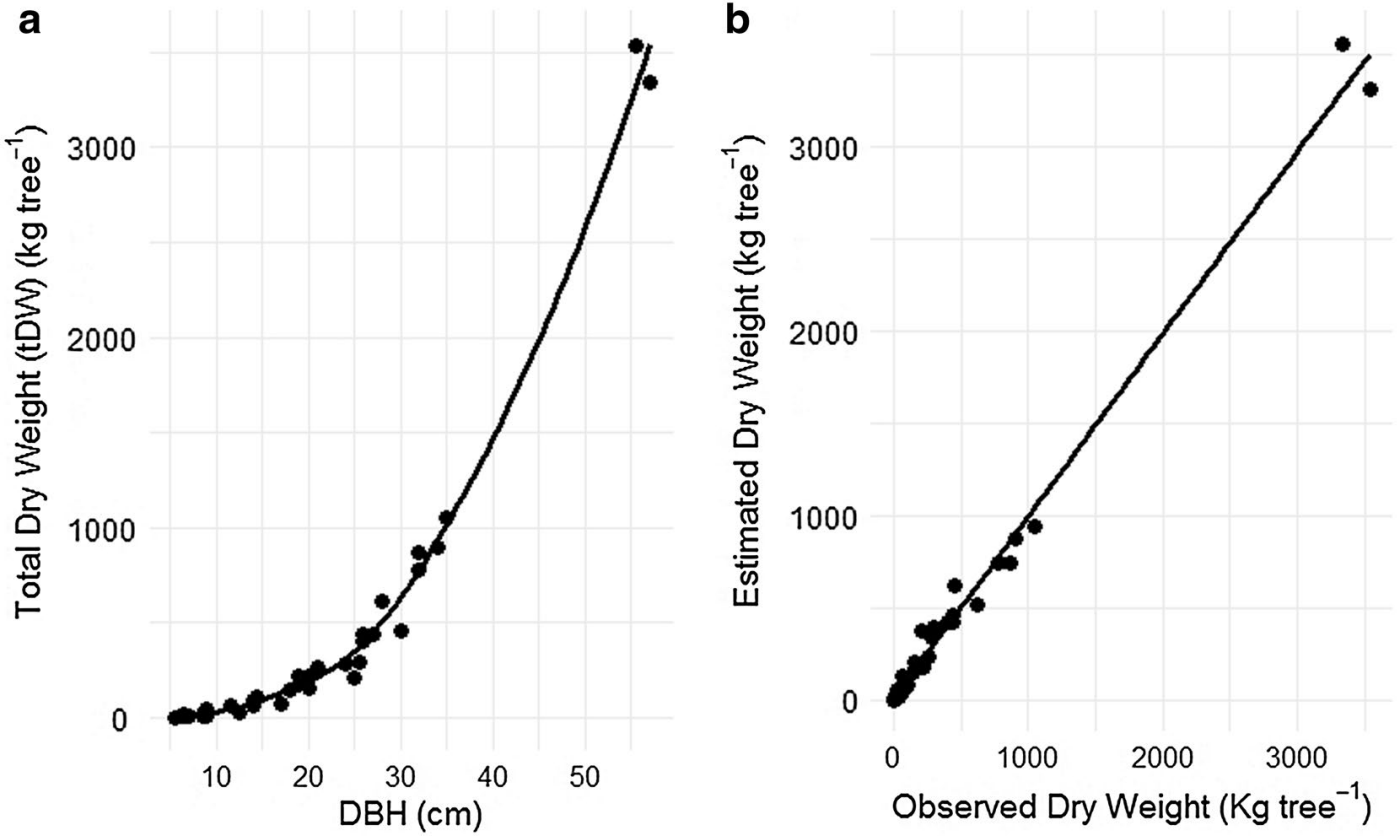
**a** Relationship between total above-ground tree dry weight (kg) and diameter at breast height (DBH), according to the power model *tDW* = 0.0613 × DBH^2.7133^ fitted in this study; **b** relationship between estimated and observed total dry weight tested for the for the 39 trees used to fit the power model above (Y = 0.986X + 9.429, adjusted R-squared 99%, RMSE 75 kg tree^−1^, t = 61.1 and P < 0.0001, and degrees of freedom 39)

The mean AGB was determined using two interchangeable procedures (Eqs. 8 and 9) and by averaging the biomass of the 27 plots sampled in this study. At plot level, biomass was calculated based on Eq. 8, which was developed in this study (Table 3 and Fig. 2), and on Eq. 9, which uses the BEF, mean stand stem volume and *WBD*. The differences between the two methods were tested using paired samples, two-tailed Wilcoxon test as the AGB from selected model, BEF and Brown’s model do not followed Gaussian distribution.

8 $$ AGB,_{j} = \left( {\frac{0.001}{{Area_{j} }}} \right)x\mathop \sum \limits_{i = 1}^{n} \left( {tDW,_{i} } \right) $$

9 $$ AGB,_{j} = \left( {\frac{0.001}{{Area_{j} }}} \right)\,x\, V,_{j} \,x\, WBD \,x\, BEF $$

where AGB,_j_ (Mg ha^−1^) is above-ground biomass of each sampled plot j; *tDW*_*i*_ (kg tree^−1^) is total dry weight (bole stem, branches and foliage) of each individually weighed tree at each sampled plot; Area,_j_ (hectares) is the area of each sampled plot (0.25 ha); *n* is number of trees found in each plot; 0.001 is a conversion factor from dry weight (*tDW*_*i*_; in kilograms) to AGB_j_; (Mg ha^−1^); *V*_j_, (m^3^ ha^−1^) is the stand volume according to Marzoli [4]; *WBD* (Mg m^−3^) is average wood basic density; and *BEF* (unit less) is biomass expansion factor.

## Results

### Adjusted biomass allometric equation

Parameter estimates and model performance criteria are presented in Table 4. The model 1 with both *DBH* and *TH* and model 2 with *DBH*, *TH* and *WBD*, are those with lower AIC value amongst all fitted models, with 436 and 438 respectively (Table 4). The same models performed better in RSE value with 61 and 62 kg tree^−1^ for model 1 and 2, respectively. However, based on MPE and RMPE, model 4 was the best performing with only *DBH*, but all models generated RMPE but were not statistically significant (t test, P > 0.05). Model 4 had AIC value (454) and RSE (77) almost the same as model 3 (AIC = 453, RSE = 75) with *DBH* and TH as independent variables. All models had negative RMPE values, which means that they are overestimating the *tDW*. Models 5 and 6 are the ones with the highest values of all computed statistics of goodness of fit (AIC, RSE and RMPE). *WBD* coefficients in model 3 and 2 were not significantly different from zero (t test, P > 0.05, Table 4). Adding *TH* and *WBD* as a predictor variables to a model with *DBH* did not improve the performance of the models. Thus, based on AIC, models 1 or 2 were the best than model 4, but given that MPE and RMPE were slightly lower for model 4, this can be shortlisted as candidate best-fitted model (with MPE = − 4.3 kg tree^−1^ and RMPE = − 1.1%).

**Table 4:** Parameters estimated and statistics of the six candidate regression functions tested to predict total dry weight (*tDW*) of the moist evergreen forest in MFR in Mozambique

Parameter	Alternative biomass allometric models					
	Model 1^ab^	Model 2^b^	Model 3	Model 4^bc^	Model 5	Model 6
AIC	436	438	453	454	533	540
RSE (kg tree^−1^)	61	62	75	77	213	233
MPE (kg tree^−1^)	− 4.7	− 4.9	− 5.1	− 4.3	− 17	− 21.4
RMPE (%)	− 1.2	− 1.2	− 1.3	− 1.1	− 4.3	− 5.4
b_0_	0.0912***	0.0969***	0.0865**	0.0613***	0.0941^ns^	0.0441^ns^
95% conf. inter. of b_0_	(0.0603 to 0.1359)	(0.0572 to 0.1597)	(0.0469 to 0.1533)	(0.0378 to 0.0963)	(0.0281 to 0.2716)	(0.0109 to 0.1483)
b_1_	2.8131***	− 0.2612***	2.6416***	2.7133***	0.9608***	1.0112***
95% conf. inter. of b_1_	(2.7135 to 2.9166)	(− 0.3827 to − 0.1398)	(2.5070 to 2.7857)	(2.5983 to 2.8358)	(0.8599 to 1.0750)	(0.8978 to 1.1385)
b_2_	− 0.2698***	2.7945***	0.3057^ns^			
95% conf. inter. of b_2_	(− 0.3816 to − 0.1583)	(2.6585 to 2.9391)	(− 0.0512 to 0.6786)			
b_3_		0.0596^ns^				
95% conf. inter. of b_3_		(− 0.2468 to 0.3748)				

*TH* is total height, *RSE* is residual standard error, *AIC* is Akaike’s information criterion, *b*_*0*_ *and b*_*1*_ are the regression coefficients

^a^ Equation that fitted better to the data, based on lowest RSE and AIC values

^b^ Equation selected for further analysis

*** significant at α = 0.001, ** significant at α = 0.01, * significant at α = 0.05, *ns* not statistically significant at α = 0.05

**Fig. 3:**
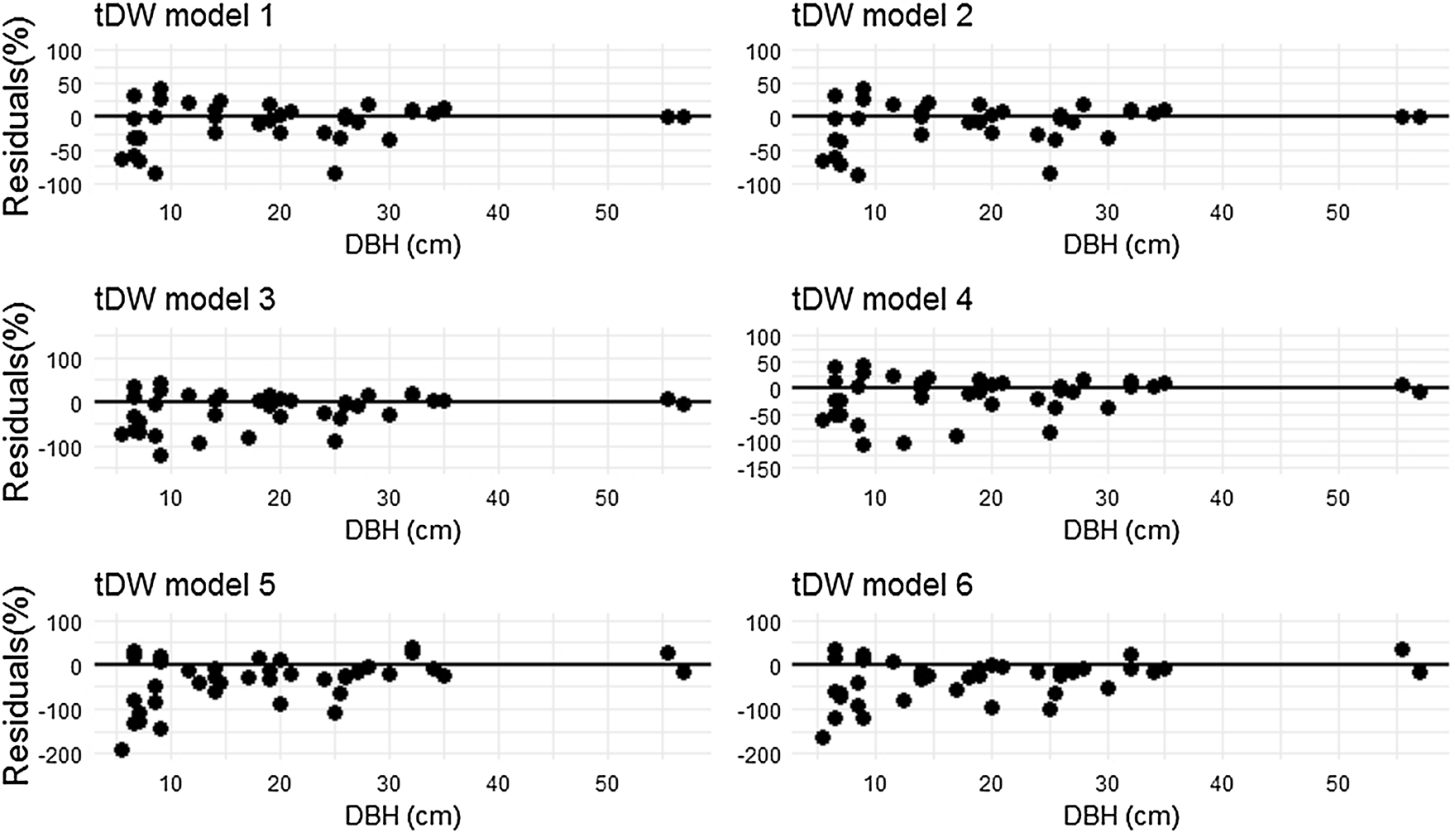
Relative residuals in the prediction of total aboveground biomass versus *DBH* for 39 trees in MFR

The model 1, 2 and 4 were selected as the best fits. However the model 4 with only *DBH* is a candidate for AGB estimation in this study because its variables is in agreement with our data from forest inventory, and it adequately describes the relationship between *tDW* against *DBH* (Fig. 2a). The diagnostic of the assumption of linearity between estimated and observed dry weight showed a satisfactory degree of statistical credibility to justify its use (Fig. 2b). So, the slope of the regression line was significantly different from zero (t test, P < 0.0001), which justifies the use of the BAE herein proposed for AGB estimation in the moist evergreen forest of MFR. The residual distribution of each model is presented in Fig. 3, which suggests that model 1, 2, 3 and 4 are all inaccurate but are precise as the scatter dots slightly shifted from zero line, while model 5 and 6 are inaccurate and not precise, as shown in Table 4 and Fig. 3.

### Comparison with existing biomass allometric equations

**Fig. 4:**
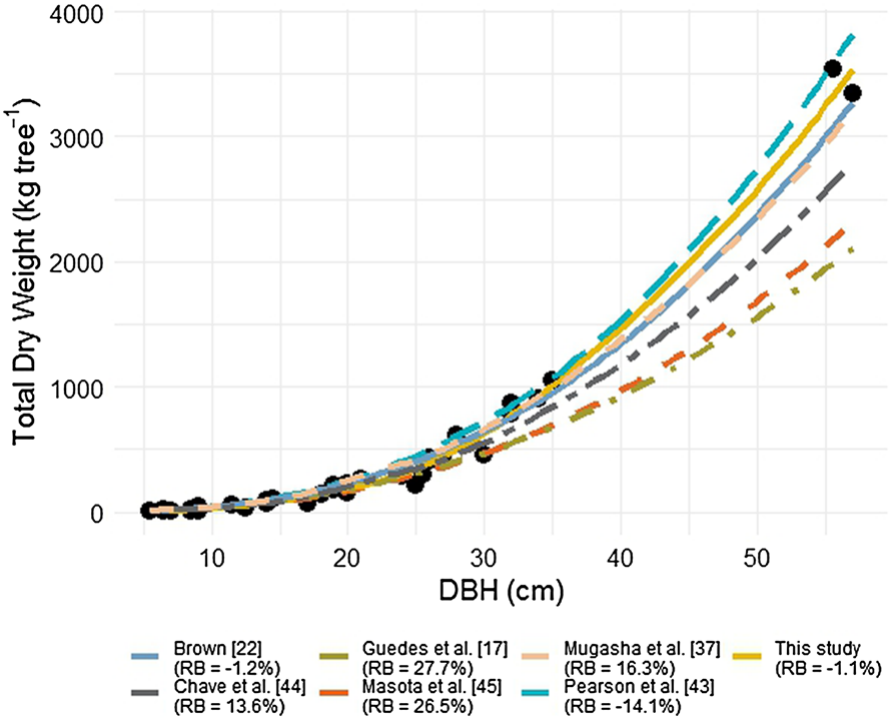
Graphical visualization of the predictive accuracy of the allometric model developed in this study against those selected from the literature, as compared with observed dry weight

The BAE found in the literature (Table 3) and tested in this study predicted *tDW* of sample trees with bias ranged from − 1.2 to 55.9% (Table 5 and Fig. 4). The equation from Brown [22] gave good estimates of AGB at MFR (RMPE = − 1.2%). All BAE developed for miombo woodlands underestimated *tDW* with large difference, and high bias were found to Guedes et al. [17] with 27.7%. Mugasha et al. [37] from miombo woodland underestimated *tDW* of moist forest of MFR with 16.6% bias. Pan-tropical models of Pearson et al. [41] and Chave et al. [42] had almost the same performance in terms of bias of estimation of *tDW* with − 14.1% and 13.6%, respectively. However, Chave et al.’s model underestimate the *tDW*, while Pearson et al. [41]’s model overestimate *tDW* of moist evergreen forest of MFR, although the bias was not statistically significant (t test, P > 0.05). However, Fig. 3 indicates that Pearson’s model is well fitted to the sample data as showing the same trend with Brown’s trend line below, beside selected model of this study (model 4). All miombo woodland models are extremely far from trend line of this study. Masota et al. [43]’s trend line is slightly beside all trend line of miombo woodland models (Fig. 4). Thus, based on the results of Table 5 and Fig. 3, Pan-tropical Brown [22]’s model is well fitted to our sample data, and miombo woodland models are not adequate to use in the moist evergreen forest of MFR.

**Table 5:** Predictive accuracy of the *DBH-*based model developed in this study against the models selected from the literature, as compared with observed *tDW*

Biomass allometric equation	MAE (kg tree^−1^)	RMPE (%)
This study (model 4)	− 4.2	− 1.1
Brown et al. [22]	− 4.9	− 1.2
Pearson et al. [41]	− 56.2	− 14.1
Guedes et al. [17]	110.2	27.7
Mugasha et al. [37]	64.8	16.3
Chave et al. [42]	54.2	13.6
Masota et al. [43]	105.5	26.5

### Diameter–height models developed

**Fig. 5:**
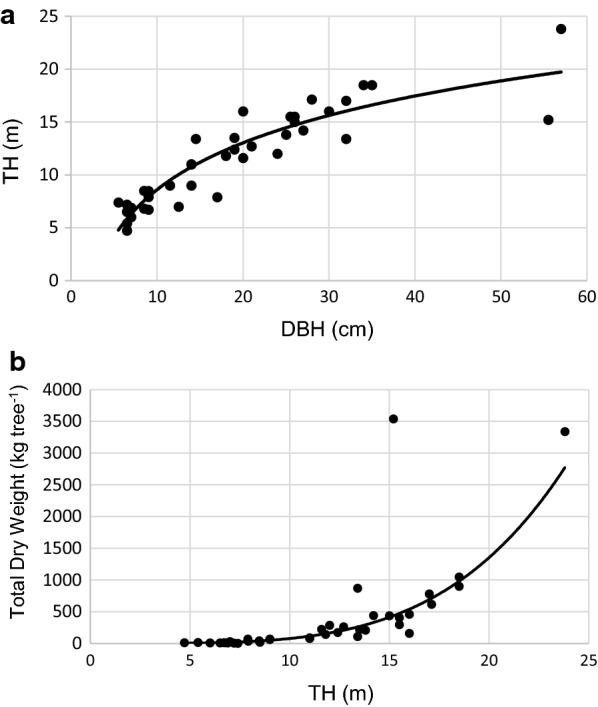
Relationship between *TH* and *DBH* (**a**) and relationship between *TH* and *tDW* (**b**) of sampled tree data

**Fig. 6:**
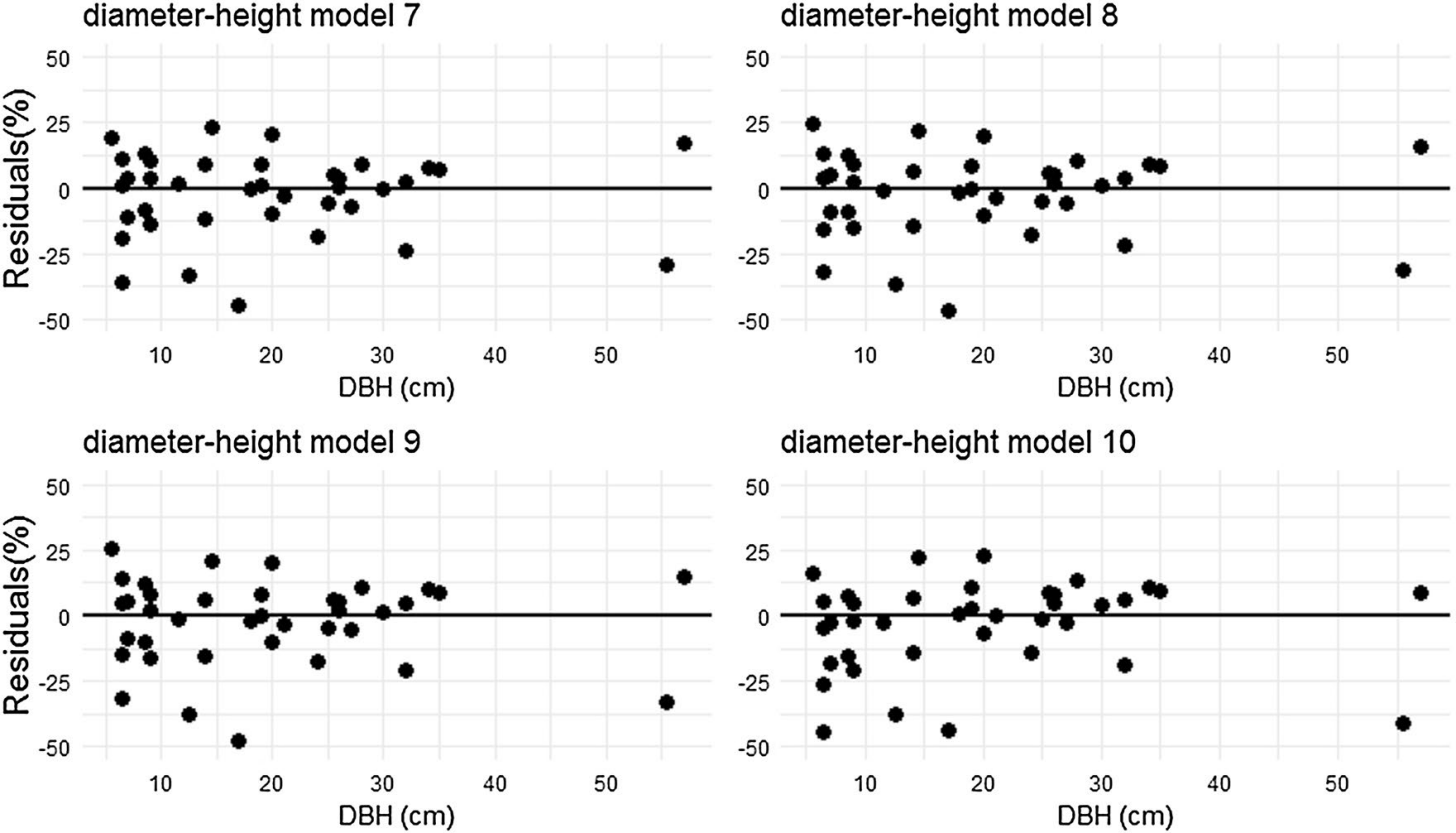
Relative residuals in the prediction of *TH* versus *DBH* for 39 trees in MFR

The results of height–diameter models performance is presented in Table 6. The height–diameter models suggested that the *TH* has high correlation with *DBH* (*r* = 0.88, P < 0.001), which indicates that more than 80% of variability in *TH* is explained by *DBH* (Adj. R^2^ = 84%, Table 6). The height–diameter model 7 had the lowest AIC and RSE and the power height–diameter model 10 had higher AIC and RSE value amongst all models (Table 6). Model 8 had the lowest MAE and RMPE amongst all tested models, making it the best-fitted model. The estimates of parameters of height–diameter model 8 (Mitscherlisch model) were b_0_ = 22.4 m (± standard error: 2.8 m) for the asymptotic height, b_1_ = 20.8 m (± 2.1 m) for difference between asymptotic and minimum height, and b_2_ = 0.039 cm^−1^ (± 0.012 cm^−1^) for the shape parameter and had the same AIC value (161) with height–diameter model 9. The asymptotic height reported by model 8 can also be shown in Fig. 5a, which reached the asymptotic height of 20 m (very close to 22.4 m from selected model), and the *DBH* reached 45 cm. However, Fig. 5b is showing that *tDW* is still increasing as a result of tree growth in diameter. Figure 5a is suggesting that our sampled data covered all range of total tree height of moist forest of MFR. All diameter–height models had good performance based on residuals distribution showed in the Fig. 6.

**Table 6:** Parameter estimated of height–diameter relationship with Mitscherlisch model as the best-fitted

Parameters	Diameter–height models tested			
	Model 7	Model 8	Model 9	Model 10
AIC	160	161	161	163
RSE (kg tree^−1^)	0.84	0.84	0.83	0.82
Adj. R^2^	1.79	1.8	1.81	1.87
MPE (kg tree^−1^)	3.17E−04	1.17E−09	1.50E−03	− 0.03
RMPE (%)	2.72E−03	1.00E−08	0.01	− 0.26
b_0_	19.1887***	22.4381***	0.4833^ns^	2.4988***
95% conf. inter. of b_0_	(16.3862 to 23.6319)	(18.4177 to 32.4790)	(− 3.8488 to 6.0111)	(1.8953 to 3.2535)
b_1_	2.0685***	20.7766***	1.0296***	0.5346***
95% conf. inter. of b_1_	(1.6784 to 2.6690)	(17.2706 to 29.0343)	(0.4104 to 1.5866)	(0.4519 to 0.6192)
b_2_	0.0690***	0.0383**	0.0343***	
95% conf. inter. of b_2_	(0.0453 to 0.1011)	(0.0179 to 0.0649)	(0.0211 to 0.0495)	

*** significant at α = 0.001, ** significant at α = 0.01, * significant at α = 0.05, *ns* not statistically significant at α = 0.05

### Biomass expansion factor determined

**Fig. 7:**
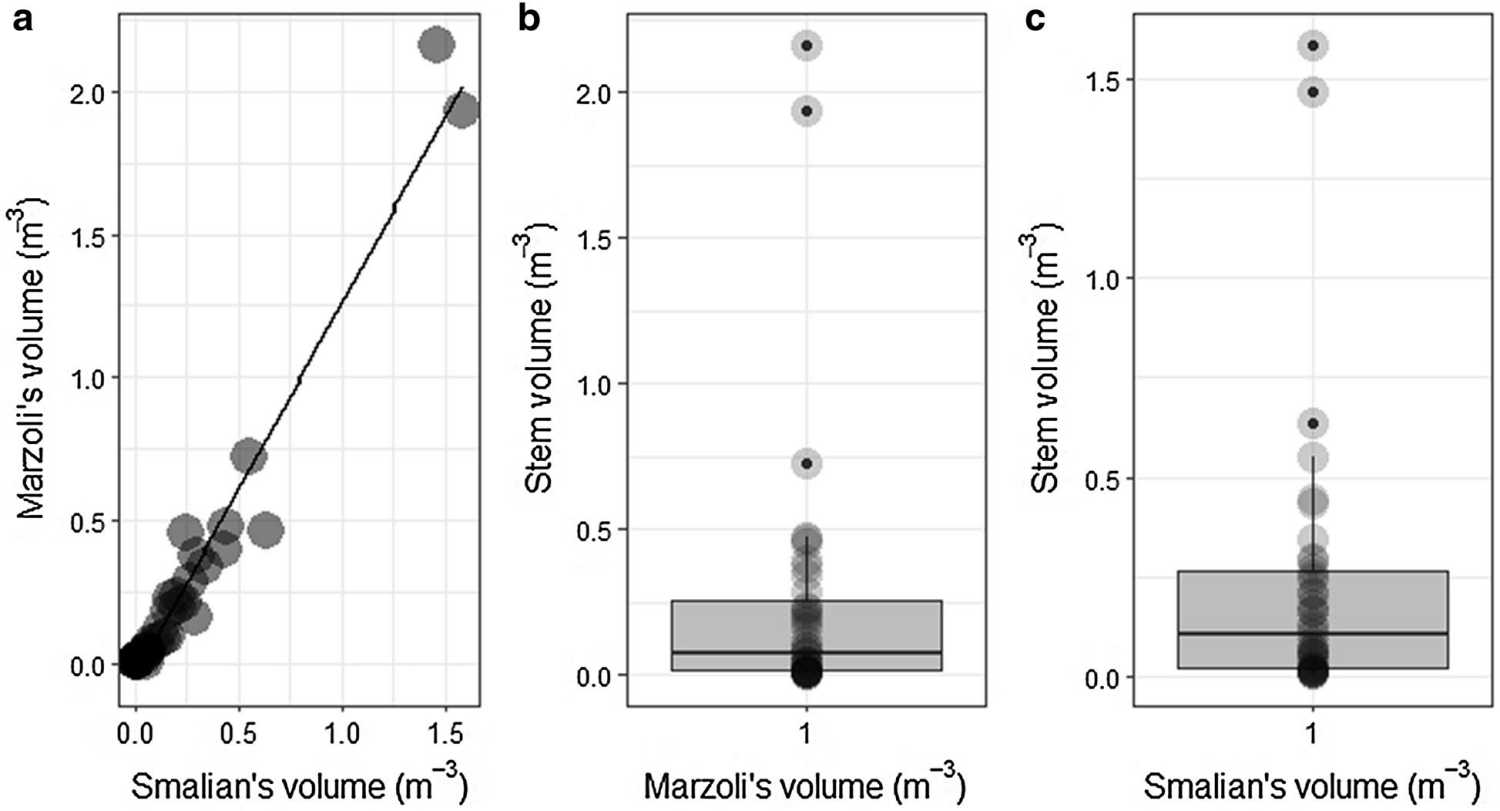
**a** Relationship between Smalian’s volume and Marzoli [4]’s volume (Y = − 0.0453 + 1.304X (Y = 1.304 − 0.0453, R-squared 96%, RSE 0.091 m^−3^, P < 0.0001, and degrees of freedom 37), and (**b**, **c**) descriptive statistics and mean comparison between Smalian’s volume and Marzoli [4]’s volume generated from sampled trees of MFR. The volume estimated using Smalian’s equation and Marzoli’s equation did not differ significantly between each other (paired samples, two-tailed, Wilcoxon test, P > 0.05)

The average BEF established for the moist forest of MFR was 2.05 ± 0.15 (mean ± standard error), and the general *WBD* suggested for all species of the studied forest was 0.715 ± 0.018 g cm^−3^. The stem volume, as depicted in Fig. 7, did not differ significantly than the one estimated by the Smalian’s formula (paired sample, two-tailed, Wilcoxon test, P > 0.05). Therefore, in the following analyses, this study uses the merchantable volume estimated using Marzoli [4]’s equation to harmonize with the general procedures currently used to estimate stem volume in national and sub national forest inventory reports in Mozambique. The mean merchantable volume estimated from Marzoli [4]’s equation was about 244.84 ± 79.63 m^3^ ha^−1^ (mean ± 95% of confidence level).

### Estimations of above-ground biomass

Total AGB of the mountain moist evergreen forest of MFR, calculated using the BAE fitted in this study, averaged 290.73 ± 140.80 Mg ha^−1^ (mean ± 95% of confidence level), and the one calculated by the BEF averaged 386.77 ± 125.79 Mg ha^−1^. The BEF method generated significantly higher AGB than biomass model (Wilcoxon test, two-tailed, P < 0.0001). The AGB estimated using Brown’s model is somehow providing additional evidence that Brown’s model is still applicable to the MFR, and estimated about 247.67 ± 104.83 Mg ha^−1^ of AGB, which was not significantly different with the AGB estimated by the selected biomass model of this study (Wilcoxon test, two-tailed, P > 0.05).

## Discussion

### Biomass allometric equation

The BAE was developed for mountain moist evergreen forest in MFR as a tool of biomass estimation and hence, carbon stocks and emissions in Mozambique. The selected BAE (model 4) was consistent with several authors that fitted the same power model with only *DBH* as a predictor variable [10, 15, 22, 37, 44, 46, 47]. The selected model does not include the tree height as a predictor variable, although some studies indicated that including tree height as predictor can improve the performance of model [38, 48, 49]. However, our results showed that the model with both *DBH* and *TH* (model 1 and 6) did not improve significantly its performance in comparison with model 4 with only *DBH*.

**Fig. 8:**
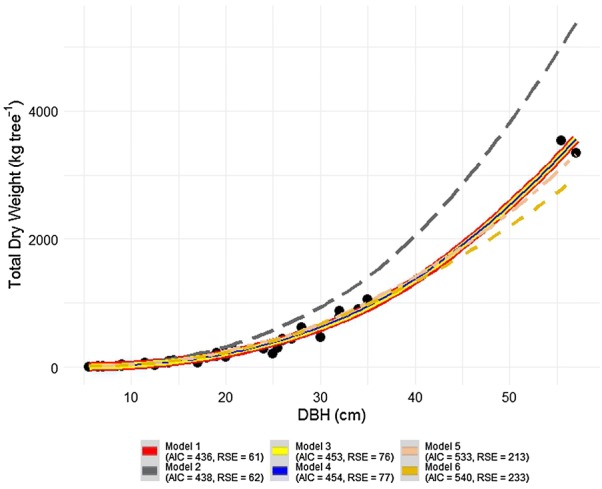
Comparison between model with only *DBH* as predictor variable and model with *DBH*, *TH* and *WBD* as predictor variables. The *blue* line of selected model (model 4 with *DBH* as predictor variable) overlap with *red* line (model 1 with *DBH* and *TH* as predictor variables) and with *yellow* line (model 2 with *DBH*, *TH* and *WBD* as predictor variables)

The most important predictor of volume or AGB is usually *DBH*. Depending on the desired precision and availability of additional predictors, a measurement of height, *WBD* and an higher diameter can also be included if they significantly reduce the volume prediction error [33]. All tested models indicated that using *DBH*, *TH* and *WBD* with four model parameters had good performance just based on AIC. Adding *TH* and *WBD* as a predictor variable to a model with *DBH* did not improve the performance of the models. This can be seen in Fig. 8, which shows that model 2 with *DBH*, *TH* and *WBD* as predictors overlapped to model 4 with only *DBH*, both models generate *tDW* which falls into the same confidence interval of 95%.

According to Ebuy et al. [50], a model which depends on *DBH* only is more appropriate when dealing with forest inventory data. The models (1–6) fitted in this study are commonly used and fitted by researchers [22, 37, 44, 46, 47], however, we selected the model with *DBH* rather than model 2 with *DBH*, *TH* and *WBD* as both models had the same performance. According to Backer et al. [51], *WBD* is an important predictor of stand-level AGB, meanwhile, Overman et al. [52], in their study, recommend using the model with *DBH* only even with the lower goodness of fit relative to the other models fitted with *DBH* and *WBD* as predictor variables. This argument has been supported by Ebuy et al. [50], who stated that *WBD* becomes useful when extensive database exists. In this case, *WBD* can be used with model 6 to predict AGB of moist evergreen forest of MFR. However, it does not significantly bring any significant added value in accuracy to estimate AGB of the mountain moist evergreen forest, when compared to the single variable model (model 4) selected as the best model that deal with our forest inventory data. Yet, model 2 instead of *DBH* and *WBD* has also *TH* as predictor, and *TH* is very difficult to assess in closed canopies of MFR. Moreover, the conventional data collection in forest inventory in Mozambique usually estimates, rather than measures, total tree heights. Tree heights in many cases are not recorded in the closed forest such as mountain moist evergreen forest of MFR where the access to canopy is difficult [53].

Where total height is particularly needed for other purposes than estimation of AGB, the diameter–height model 8 (Table 6) developed in this study for *TH* estimation can be used. For instance, Chave et al. [38] have used tree height model to estimate AGB in pan-tropical moist forest. However, Feldpausch et al. [54] stressed that the integration of tree height into BAE underestimates carbon storage by 13%.

### Model comparison with existing models

Pan-tropical models from Pearson et al. [41], including the allometric model developed by Guedes et al. [17] for a miombo woodland of Mozambique, were compared to our selected model (model 4) which showed differences between two sites. The other pan-tropical model that is mostly used, developed by Chave et al. [42], was compared in this study and it had bias of 13%, suggesting different climatic conditions from the region where it was developed. The relative mean prediction error (RMPE) of Brown [22]’s model of moist tropical forest showed that it can be applicable to predicting AGB in our study area despite not having included tree species from Africa. All models of miombo woodlands [17, 37] and moist forest [43–43] were not applicable to estimate AGB at the mountain moist evergreen forest in Mozambique.

While Brown [22]’s model underestimates somehow the dry weight of trees larger than 35 cm, it generates estimation with the relative bias of 1.2% (*P* < 0.05) close to those generated by the best-fitted model of this study with the relative bias of 1.1% (*P *> 0.05). On the other hand, Pearson’s model estimated lower AGB with a relative bias of 26.60% (*P *< 0.01). Differences between BAE could also result from regional differences in diameter–height allometry [55]. The tree allometry could be the key source of the differences found, as mountain moist evergreen forest are typically tall trees that may grow much more than 20 m, while lowland miombo is dominated by shorter trees for the same diameter, which justifies lower dry weight for trees in these woodlands. Eventually, the allometry of the trees in the Brown’s sample may have included trees with similar allometry to the trees of our study area, although Brown’s model consistently predicted lower AGB than our selected model. Gibbs et al. [13] argued that the effort required to develop species or site-specific BAE would not typically improve accuracy in AGB estimations. Contrary to the results of Gibbs et al. [13], our results on existing model performance is in part in agreement with what was found by Ngomanda et al. [12], who stated that the pan-tropical equations currently do not correctly capture the variability of biomass allometry at the global scale. This can be seen with tested pan-tropical models (Pearson et al. [41]’s model and Chave et al. [42]’s model, Table 5 and Fig. 4).

### Estimation of the biomass expansion factor

The average *WBD* found in this study can be comparable with those found in lowland tropical rain forest in Costa Rica (range from 0.27 to 0.74 g cm^−3^) [36]. Muller-Landau et al. [36] indicate that for biomass calculations, site average *WBD* values should ideally be weighted by wood volume. The values of BEF found in this study were not statistically different from those obtained by Machoco [16], for lowland miombo woodlands of the Central Mozambique, with values ranging from 1.20 to 5.09 and averaging 2.03 ± 0.14. We would expect BEF from mountain moist evergreen forests to be lower than that of lowland Miombo woodlands. However, the lack of differences can be due to the data sources. Machoco [16] used average *WBD* obtained from the literature while in this study we used direct measurements through destructive sampling.

The BEF obtained in this study was much smaller compared to the moist central African forest reported by Ngomanda et al. [12] with 1.55 (range: 1.04 and 5.59), obtained by Segura and Kaninnen [23] in tropical humid forest of Costa Rica (mean 1.60 ± 0.20 ranging from 1.4 to 1.9), and reported by Henry et al. [45] in Ghana (mean 1.51, range: 1.13 to 2.20) or by Djomo et al. [48] in southeast Cameroon (mean 1.22, range: 1.02 to 2.02). This is probably because of differences in biomass allocation among different tree components, in diameter–height tree allometry and the crown architecture in different regions [12, 55]. The BEF estimate at MFR was lower than the value of 3.4 reported by the IPCC for tropical forest stands, but it was consistent with that found by Brown and Lugo [2] with an average range from 1.1 to 2.5, obtained from the forests of Africa, America, and Asia. Brown [56] states that tropical forests tend to have higher BEF for a given volume and tree size reflecting the large size of the tree crowns when compared to those of temperate forests. Brown et al. [24] estimated different BEF for primary, secondary and non-productive rainforest of Sri Lanka, and presented average values around 2.02, 2.26 and 4.48, respectively. According to those values, the BEF found in this study is within primary to secondary forest, perhaps because of high level of human disturbance which makes the forest in MFR more of a transition mountain moist evergreen forest with typically large crowns than in an undisturbed forest.

### Estimation of above-ground biomass

The models of above-ground tree biomass (AGB) in this study were developed using data with *DBH* range from 5 to 57 cm. However, according to the forest inventory data, the maximum *DBH* of the trees recorded in the study area was 179 cm. Thus, including individual trees with bigger size than 57 cm would be necessary in the future to ensure the representativeness of individual’s trees in the ecosystem. The study was carried out in a conservation area where national legislation does not allow logging activities. With this limitation, the sample trees were cut in the buffer zone of MFR, where human disturbance is high. Consequently, there was lack of large size trees (*DBH* > 40 cm), and this was also recorded during the forest inventory inside the protected area.

**Fig. 9:**
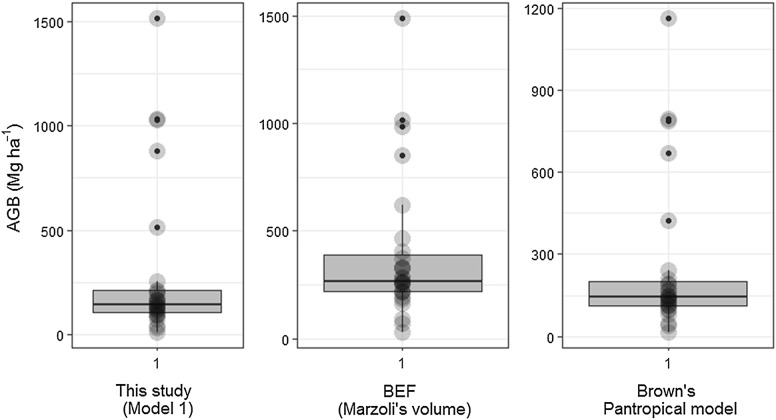
Above-ground biomass estimated by selected biomass allometric model and BEF of this study as well as Pan-tropical model of Brown [22]

The fitted models should be used with caution with large trees (*DBH* > 57 cm) because they can overestimate the AGB. In this sense, a comparison of AGB was made between the model of this study and Brown’s for mountain moist evergreen forest, however, there is no significant difference between AGB estimates from the two models (Fig. 9). This indicates that the BAE of this study estimates AGB as expected for moist evergreen forest, regardless of the size of the trees of fit. It should be noted that both models generate over-estimate total above ground tree dry weight for larger trees (*DBH* > 100 cm), as it can be seen in Fig. 9, where all plots with trees larger than 100 cm became an outlier, trees with *DBH* > 100 cm accounting for 39% of the AGB, 18 trees with *DBH* above 100 cm, 33 trees with *DBH* ranging from 60 to 100 cm, 2634 trees with *DBH* < 60 cm, and 2685 trees were measured in inventory forest.

The main purpose of Marzoli’s volume equation application is to explore the possibility of using the volumes table from the national forest inventory generated by Marzoli [4] so that they are used to convert AGB through the BEF. Figure 7 shows that the Smalian’s volume was not different with Marzoli’s volume of sampled trees (paired sample, two-tailed, Wilcoxon test, *P* > 0.05). Thus, the volumes table of Marzoli [4] can be converted to AGB of the forest of the study area using the BEF developed in this study, as shown in Fig. 9 below. Here, there is enough evidence that the BEF overestimates the AGB of the study area and BAE becomes more accurate than BEF.

The mean values of AGB obtained in this study for model 4, BEF and Brown [22]’s model had high variability among plots, as seen for large confidence intervals. Many factors can explain the variability of AGB [heterogeneity of landscape (mature stand forest and secondary forest), soil fertility, wood basic density, high diameter and other factor] [51, 54, 55]. In general, the AGB estimated from selected model 1 (290.73 Mg ha^−1^) and from the developed BEF (386.77 Mg ha^−1^) of this study indicated that the mountain moist evergreen forest stores up to four times more carbon than miombo woodlands of southern Africa lowland, and in agreement with Desanker et al. [57], who state that the AGB in dry miombo woodland is usually low, and is about 55 Mg ha^−1^ (ranging between 21 and 84 Mg ha^−1^), while for wet miombo woodland it is about 90 Mg ha^−1^, ranging between 44 and 144 Mg ha^−1^.

The mean AGB estimated with BEF overestimates the AGB of the study area, in part, because of the overestimation observed with Marzoli’s volume. Moreover, the AGB from BEF is within the range reported by Maniatis and Mollicone [58] in three forest types in the Congo Basin forest (AGB ranging from 312 to 333 Mg ha^−1^). Munishi and Shear [35] reported an AGB of about 1055 ± 35 Mg ha^−1^ and 790 ± 20 Mg ha^−1^, for the evergreen mountain undisturbed forests of Usambaras sand Ulugurus Forest Reserves (highlands, MAP ~ 2900–4000 mm and lowlands with MAP ~ 1200–3100 mm) respectively, in Tanzania. These values are higher than those found in this study, which could be related to the higher level of human disturbance in MFR. According to Ryan et al. [8], human activities such as agriculture, charcoal production, and timber collection are responsible for about 46 and 56% of total biomass loss in Sussundenga District, where MFR is located.

## Conclusions

The main objective of this study was to develop a BAE and BEF for AGB estimation in mountain moist evergreen forest of MFR in Mozambique as a step forward for REDD+. The power model with only *DBH* was selected as the best fit for the whole tree dry weight (*tDW*) of mountain moist evergreen forest of MFR. This model presented a combination of lower RMSE, although slightly high AIC compared to the alternative candidate models evaluated. In contrary to global model [42, 59], *WBD* did not improve the performance of models in all tested models. Total height seems to be a powerful predictor variable when combined with *DBH*, but it had at least the same statistical performance with the model with only *DBH*. Thus, we recommend the model with only *DBH* as predictor instead of that with *DBH*, *TH* and *WBD* together. The comparison using global models showed that the selected model for this study was more accurate for trees sized out of the fit data, suggesting that the selected model is reliable and can be used to estimate AGB in the study area with the same level of accuracy as the global model, and it has potential to be applied in other mountain regions in Mozambique, where mountain moist evergreen forests occur. However, our model could be improved with more data, particularly with larger size trees, but such trees are protected by the law of conservation areas in Mozambique. The BEF of this study can be applied to predict AGB through converting the volume table from national forest inventory developed by Marzoli [4], however, BEF overestimate the AGB. This study suggests that despite being a disturbed conservation area, the MFR still has a large stock of carbon comparable with the mountain moist evergreen forest in the world, but with significant variability of AGB between plots. Therefore, mountain most evergreen forest has a huge potential to provide financial resources through C-based payment for ecosystem services under REDD+ mechanism.

## Abbreviations

BAE: biomass allometric equation
REDD+: Reduction of Emission from Deforestation and Degradation
BEF: biomass expansion factor
AGB: above-ground tree biomass
*DBH*: diameter at breast height
t*DW*: total tree dry weight
*TH*: total height
